# Seroprevalence of *Toxoplasma gondii* in Pregnant Women and Bioassay of IgM Positive Cases in Zanjan, Northwest of Iran

**Published:** 2012

**Authors:** F Hajsoleimani, A Ataeian, AA Nourian, S Mazloomzadeh

**Affiliations:** 1 Department of Parasitology and Mycology, Zanjan University of Medical Sciences, Zanjan, Iran; 2 Department of Social Medicine, Zanjan University of Medical Sciences, Zanjan, Iran

**Keywords:** *Toxoplasma gondii*, Prevalence, Pregnant women, ELISA, Iran

## Abstract

**Background:**

The aim of this study was to determine the *Toxoplasma* antibodies in pregnant women in Zanjan, by ELISA method.

**Methods:**

Blood samples were taken from 500 pregnant women referred to the health centers of Zanjan City, North West Iran, IgM and IgG titers were primarily evaluated. The collected data were analyzed with SPSS 11.5 using Chi-Square test.

**Results:**

Anti *Toxoplasma* IgM and IgG were positive in 1.4% and 37.2% respectively. Seropositive subjects were more frequently seen in those with >30 years old compared to younger women (<20 years old). No significant relationship was found between the seroprevalence of *T. gondii* infection and level of education, residence area, history of abortion and gestational age.

**Conclusion:**

The rate of IgM positive was low; however, a large number of the studied population were IgG positive, indicative of having a latent infection due to the past exposure to *Toxoplasma* parasite in this region.

## Introduction

Toxoplasmosis is a worldwide zoonosis that can infect approximately one third of the world's population by a protozoan parasite, *Toxoplasma gondii*. However, it may appear in several forms, infections in humans are usually clinically unapparent, and latent infection usually persists for life, which is not seen as a health threat except in case of pregnancy and immunocompromised ind-ividuals. *Toxoplasma gondii* can be transmitted to humans by three principal ways; first, by eating raw or uncooked meat, especially pork, mutton or uncooked food and those who are in contact with infected meat. Second, by consuming oocysts those cats have passed in their feces, either from a trash box or from soil (e.g., soil from gardening, or unwashed fruit or vegetables, or in unrefined water). Third, by transmitting the infection from infected pregnant women to their unborn fetus it can be happened. It is known that the parasite can cause congenital disease and abortion both in humans and livestock.

In most cases the laboratory diagnosis of acute and latent toxoplasmosis relies on the detection of *T. gondii* specific IgG and IgM antibodies and the avidity test of *T. gondii* specific IgG antibodies has also been very helpful in the diagnosis. Many serological tests such as the latex agglutination test, ELISA, indirect fluorescence antibody test (IFA) and hemagglutination test have been used in the detection of antibodies against *T. gondii* in pregnant women (1, 2).

There are many studies on the prevalence of anti-*T. gondii* antibody among Iranian women. Seropositivity of *T. gondii* is 48%-74.6% in northern areas (3–5), 33%-44% in northwest (6–10), 22%-37% in south (11–13) and 27%-54% in central parts of Iran (14–17). The seroprevalence of the infection in pregnant women in Zanjan was reported as 17.9% (18, 19).

The present study was performed to determine the *Toxoplasma* antibodies in pregnant women in Zanjan, by ELISA method because of its high sensitivity and specificity, easier technique and lower expanse which is preferred in order to screening *Toxoplasma* infection.

## Materials and Methods

This study was carried out in Zanjan City, North West Iran; which has an area of 36,400 km^2^. Zanjan has a highland climate characterized by cold snowy weather in the mountains and moderate climate in the plains in winter time. In the summer, the weather is warm. The average maximum temperature of Zanjan is around 27 °C, whereas the average minimum temperature stands at -19 °C.

### Patients and blood sampling

Three ml of venous blood sample were drowning from the study group (500 pregnant women) and anticuagulated with citrated sodium. Two ml of citrated blood were centrifuged at 3000 rpm for 15 minutes. The plasma was isolated and stored at 4 °C until the assays. Serum samples were tested for anti *Toxoplasma* IgM and IgG antibodies using *Toxoplasma* IgM ELISA kit, GD81 and *Toxoplasma* IgG ELISA kit, GD80 (Genesis Diagnostic Company/England).

The present work has been approved by Zanjan University of Medical Sciences Ethics Committee.

### Statistical analysis

The chi-square test was used to analyze the data in SPSS version 11.5.

## Results

The overall seroprevalence of toxoplasmosis in pregnant women was 37.8% (189/500 cases). IgG and IgM anti-*Toxoplasma* antibodies were positive in 186/500 cases (37.2%) and 7/500 cases (1.4%), respectively. The results, including seroprevalence data together with personal and demographic variables are detailed in Table 1. The proportion of seropositive women increased with increasing age, from 28.7% in ≤20 years old to 48% in >30 years old women (*P*= 0.025). No significant relationship was found between the seroprevalence of *T. gondii* infection and their level of education. The prevalence rate showed no significant differences between women resident in rural and those in urban areas, neither the history of abortion had significant association with *Toxoplasma* seroprevalence rate. The surveyed pregnant women at their first, second and third gestational trimesters showed indifferent rates of the *Toxoplasma* infection. The data of the above variables are summarized in Table 1.

**Table 1:** Seroprevalence of *Toxoplasma gondii* in pregnant women in Zanjan, North West of Iran

		Seropositivity		Seronegativity		*P* value
		N	%	N	%	
**Age groups (yr)**	≤ 20 years	25	28.7	62	71.3	0.025
	21-30 years	117	37.1	198	62.9	
	> 30 years	47	48	51	52	
	Illiterate	16	34	31	66	
**Education**	elementary school	91	43.8	117	56.2	0.151
	High school	33	30	77	70	
	Diploma	36	35	67	65	
	University graduated	13	40.6	19	59.4	
**Residence area**	Urban	126	38.5	201	61.5	0.643
	Rural	63	36.4	110	63.6	
**Gestational age**	1st trimester	74	43.5	96	56.5	0.157
	2nd trimester	52	35.9	93	64.1	
	third trimester	63	34.1	122	65.9	
**History of abortion**	Yes	159	37.6	264	62.4	0.819
	No	30	39	47	61	

## Discussion

A high prevalence of chronic toxoplasmosis (IgG positive) during pregnancy was found in this study (37.2%) that is accordance with weather and geological conditions of this area. In Iran, at least 30% of people have been found seropositive for anti *T. gondii* immunoglobulin G (IgG) in most regions (7). A seroprevalence of 17.9% in pregnant women referred to a hospital in Zanjan was reported (18) which was lower than what we found. The authors used indirect fluorescent antibody test (IFT), whereas we used ELISA kit; of course, that much difference between these two studies cannot be solely attributed to the different serological methods. It is noteworthy that the interval between these two studies is more than a decade. Further, the referral hospitals of the two studies were different in several aspects.

In our study, the prevalence of recently acquired infections (IgM positive) was relatively low (1.4%). The IgG seropositive rate increased with age. Women older than 30 years had a significantly higher seroprevalence (48%) compared to those who were 20 or less (28.7%) (*P*= 0.025).

Low level of education was associated with higher rate of toxoplasmosis (18–21). We did not find a significant relationship between the seroprevalence of *T. gondii* infection and the level of education. There are similar reports in Turkey and Hamadan (6, 22). Some other studies showed a significance decreases in seropositivity as the level of the education increases (7, 13, 23).

In the present study, we found no statistical difference among seroprevalence of *T. gondii* and the residence areas, which is in accordance with other reports (7, 22, 24), but some studies showed higher seropositivity in urban than in rural areas (25, 26). In addition, in the present research similar to the results of previous studies, (18, 22), no significant relationship was found between the seroprevalence of *T. gondii* infection and history of abortion in pregnant women. No significant association of the infection with gestational age was observed.

In conclusion, this study showed that exposure to *T. gondii* parasite in the past is considerable in the pregnant women in Zanjan and at the same time many of them remain susceptible to the infection and require care to avoid parasite exposure during their pregnancies.

## References

[1] Krick JA, Remington JS (1978). Toxoplasmosis in the adult: an overview. N Engl J Med..

[2] Dubey JP, Beattie CP (1988). Toxoplasmosis of animal and man.

[3] Saeedi M, Veghari GR, Marjani A (2007). Seroepidemiologic evaluation of anti-*Toxoplasma* antibodies among women in North of Iran. Pak J Biol Sci..

[4] Youssefi MR, Sefidgar AA, Mostafazadeh A, Omran SM (2007). Serologic evaluation of toxoplasmosis in matrimonial women in Babol Iran. Pak J Biol Sci..

[5] Sharif M, Ajami A, Daryani A, Ziaei H, Khalilian A (2006). Serological survey of toxoplasmosis in women referred to medical health laboratory before marriage northern Iran 2003-2004. Int J Mol Med Adv Sci..

[6] Fallah M, Rabiee S, Matini M, Taherkhani H (2008). Seroepidemiology of toxoplasmosis in primigravida women in Hamadan Islamic Republic of Iran 2004. East Mediterr Health J..

[7] Hashemi HJ, Saraei M (2010). Seroprevalence of *Toxoplasma gondii* in unmarried women in Qazvin Islamic Republic of Iran. East Mediterr Health J..

[8] Fallah M, Rabiee S, Matini M, Taherkhani H (2003). Seroepidemiology of *Toxoplasma* infection in women aged 15–45 years in Hamadan west of Iran. J Res Health Sci..

[9] Mansoori FL (2004). Seroepidemiology of toxoplasmosis in Kermanshah province west of Iran 2002. Behboud..

[10] Abdi J, Shojaee S, Mirzaee A, Keshavarz H (2008). Seroprevalence of Toxoplasmosis in Pregnant Women in Ilam Province Iran. Iranian J Parasitol..

[11] Sharifi Mood B, Hashemi Shahri M, Salehi M, Naderi M, Naser Poor T (2006). Seroepidemiology of *Toxoplasma* Infection in the Pregnant Women in Zahedan, Southeast of Iran. J Res Health Sci..

[12] Fouladvand M, Barazesh A, Naeimi B, Zandi K, Tajbakhsh S (2010). Seroprevalence of toxoplasmosis in high school girls in Bushehr city South-west of Iran 2009. Afr J Microbiol Res..

[13] Foladvand M, Jaafary SM Prevalence of antibodies to *Toxoplasma gondii* in pregnant women of Bushehr. Iranian South Med J.

[14] Manouchehri Naeini K, Deris F, Zebardast N (2004). The immunity status of the rural pregnant women in Chaharmahal and Bakhtyari province against *Toxoplasma* infection 2001–2002. J Shahrekord Med Sci..

[15] Keshavarz valian H, Nateghpour M, Zibaee M (1998). Seroepidemiology of toxoplasmosis in Karaj Iran in 1998. Iranian J Publ Health..

[16] Keshavarz Valian H, Mamishi S, Daneshvar H (2000). Prevalevce of toxplasmosis in hospitalized patient of Kerman hospitals Iran. J Med Sci Kerman..

[17] Arbabi M, Talari SA (2001). Seroprevalence of *Toxoplasma* infection in pregnant women in Kashan Iran. Feiz J..

[18] Ataeian A, Tadayon P (2000). Prevalence of *Toxoplasma gondii* antibodies in women of Zanjan Hakim Hidajy hospital. J Zanjan Med Sci..

[19] Varella IS, Wagner MB, Darela AC, Nunes LM, Muller RW (2003). Seroprevalence of toxoplasmosis in pregnant women. J Pediatr (Rio J).

[20] Nash JQ, Chissel S, Jones J, Warburton F, Verlander NQ (2005). Risk factors for toxoplasmosis in pregnant women in Kent, United Kingdom. Epidemiol Infect..

[21] Jones JL, Kruszon-Moran D, Wilson M, McQuillan G, Navin T, McAuley JB (2001). *Toxoplasma gondii* infection in the United States: seroprevalence and risk factors. Am J Epidemiol..

[22] Ertug S, Okyay P, Turkmen M, Yuksel H (2005). Seroprevalence and risk factors for toxoplasma infection among pregnant women in Aydin province, Turkey. BMC Public Health..

[23] Mostafavia SN, Ataei B, Nokhodian Z, Yaran M, Babak A (2011). Seroepidemiology of *Toxoplasma gondii* infection in Isfahan province, central Iran: A population based study. J Res Med Sci..

[24] Lebech M, Larsen SO, Petersen E (1993). Prevalence, incidence and geographical distribution of Toxoplasma gondii antibodies in pregnant women in Denmark. Scand J Infect Dis..

[25] Ades AE, Parker S, Gilbert R, Tookey PA, Berry T, Hjelm M, Wilcox AH, Cubitt D, Peckham CS (1993). Maternal prevalence of Toxoplasma antibody based on anonymous neonatal serosurvey: a geographical analysis. Epidemiol Infect..

[26] Fallahi SH, Badparva E, Mohammadi M, Ebr-ahimzadeh F, Pournia Y (2009). Ser-oepidemiological Study of *Toxoplasma gondii* in Women Referred to Khorramabad Lab-oratory of Health Center for Medical Examination before Marriage, Lorestan Province, Iran, 2007. Asian J Biol Sci..

